# Cell Structure Changes in the Hyperthermophilic Crenarchaeon Sulfolobus islandicus Lacking the S-Layer

**DOI:** 10.1128/mBio.01589-19

**Published:** 2019-08-27

**Authors:** Changyi Zhang, Rebecca L. Wipfler, Yuan Li, Zhiyu Wang, Emily N. Hallett, Rachel J. Whitaker

**Affiliations:** a Carl R. Woese Institute for Genomic Biology, University of Illinois at Urbana-Champaign, Urbana, Illinois, USA; b Department of Microbiology, University of Illinois at Urbana-Champaign, Urbana, Illinois, USA; c Frederick Seitz Materials Research Laboratory, University of Illinois at Urbana-Champaign, Urbana, Illinois, USA; Harvard University

**Keywords:** *Archaea*, cell structure, *Sulfolobus islandicus*, surface layer

## Abstract

Rediscovery of the ancient evolutionary relationship between archaea and eukaryotes has revitalized interest in archaeal cell biology. Key to the understanding of archaeal cells is the surface layer (S-layer), which is commonly found in *Archaea* but whose *in vivo* function is unknown. Here, we investigate the architecture and cellular roles of the S-layer in the hyperthermophilic crenarchaeon Sulfolobus islandicus. Electron micrographs of mutant cells lacking *slaA* or both *slaA* and *slaB* confirm the absence of the outermost layer (SlaA), whereas cells with intact or partially or completely detached SlaA are observed for the Δ*slaB* mutant. We experimentally identify a novel S-layer-associated protein, M164_1049, which does not functionally replace its homolog SlaB but likely assists SlaB to stabilize SlaA. Mutants deficient in the SlaA outer layer form large cell aggregates, and individual cell size varies, increasing significantly up to six times the diameter of wild-type cells. We show that the Δ*slaA* mutant cells exhibit more sensitivity to hyperosmotic stress but are not reduced to wild-type cell size. The Δ*slaA* mutant contains aberrant chromosome copy numbers not seen in wild-type cells, in which the cell cycle is tightly regulated. Together, these data suggest that the lack of SlaA results in either cell fusion or irregularities in cell division. Our studies show the key physiological and cellular functions of the S-layer in this archaeal cell.

## Abstract (precis)

The S-layer is considered to be the sole component of the cell wall in *Sulfolobales*, a taxonomic group within the *Crenarchaeota* whose cellular features have been suggested to have a close relationship to the last archaea-eukaryote common ancestor. In this study, we genetically dissect how the two previously characterized S-layer genes as well as a newly identified S-layer-associated protein-encoding gene contribute to the S-layer architecture in *Sulfolobus*. We provide genetic evidence for the first time showing that the *slaA* gene is a key cell morphology determinant and may play a role in *Sulfolobus* cell division or/and cell fusion.

## INTRODUCTION

The primary interface between the cell and its environment is a multifunctional cellular envelope. Comprised in this structure in some bacterial and most archaeal cells is a proteinaceous two-dimensional (2D) crystalline matrix coating the outside of the cell, called the surface layer (S-layer). Despite the broad distribution of the S-layer in cells from two domains, its generalized function has not been identified. One unifying concept suggests that the S-layer functions as an exoskeleton that interacts with other proteins in the cell membrane to coordinate diverse internal and external cell functions (1). In *Bacteria*, it has been shown that the S-layer is associated with either the peptidoglycan or the outer membrane (2), and S-layer genes are not essential for cellular viability in most studied bacteria under laboratory conditions. Individual characterization of S-layer mutants in *Bacteria* revealed that the S-layer plays highly diverse roles, serving as a protective coat or sieve, binding to specific receptors for adhesion or zones of adhesion for exoenzymes (1), maintaining cell envelope integrity (3), resisting osmotic stress (4), regulating cell morphology, and contributing as a virulence factor (5), as well as maintaining cell swimming motility (6–8). In contrast to the bacterial S-layers, archaeal S-layers are found to be the predominant, if not the sole, component of the cell wall, with very few documented exceptions (9). So far, studies of the archaeal S-layer have been limited to observational and biochemical analyses (9, 10) since its discovery in the haloarchaea Halobacterium salinarum around 60 years ago (11).

Electron microscopy-based analyses of isolated proteinaceous S-layers in archaea revealed that they are organized as a highly regular two-dimensional lattice structure that display p1, p2, p3, p4, and p6 symmetry, depending on the species (9, 12). Moreover, it has been shown that the S-layer proteins in all studied archaea undergo posttranslational modifications such as O- and N-glycosylation, with the latter type more prevalent (9, 10, 13). Currently, archaeal S-layer *in vivo* functions have not been studied extensively, but it has been proposed that the S-layer plays a role in osmotic stress (14), determines cell shape in the haloarchaeon H. salinarium (15), serves as a barrier to gene transfer in an isolated Sulfolobus islandicus population (16), and contributes to cell stability as well as cell division in the methanogen Methanocorpusculum sinense (17).

It is now well-known that the S-layer is composed of two glycosylated proteins, SlaA (∼120 kDa) and SlaB (∼45 kDa) in *Sulfolobales* (18–20). The current S-layer model in *Sulfolobus* shows a stalk-and-cap relationship between SlaA and SlaB, with SlaB as the stalk anchoring SlaA to the cytoplasmic membrane, forming a crystalline matrix that constitutes the outermost layer covering the whole cell (19). Compensating for the absence of the S-layer by forming a strong barrier at the site of cell division is hypothesized to be one role for Cdv (cell division) proteins (21). The S-layer is also believed to be a receptor for viruses and has been shown to change its structural shape after viral induction and to provide a barrier to virus egress during maturation of the Sulfolobus spindle-shaped virus (SSV) viral particle (22). Instability of the S-layer in *Sulfolobus* has been associated with changes in cell shape (23) and budding of vesicles (24, 25). It has been proposed that the archaeal S-layer assists the cell against turgor pressure (1, 9). Thus far, no generalized function for the S-layer in *Archaea* has been defined as no archaeal S-layer-deficient mutants have been characterized.

Recently, we discovered that the S-layer genes are not essential for S. islandicus M.16.4 cell survival under standard lab conditions (26). Therefore, the resulting S-layer deletion mutants provide a model system to uncover the physiological and cellular roles of the archaeal S-layer. In this study, we aim at characterizing these S-layer-deficient mutants to dissect *in vivo* functions of the S-layer in this model organism.

## RESULTS

### Isolating roles for *slaA* and *slaB* in S-layer structure and function.

As in other *Sulfolobus* species, *slaB* is located in the downstream region of *slaA* with the same orientation (see Fig. S1a available as supplemental material at FigShare [https://doi.org/10.6084/m9.figshare.8285423]) in S. islandicus M.16.4. Reverse transcription-PCR (RT-PCR) analysis showed that *slaA* and *slaB* are cotranscribed (Fig. S1b), in agreement with a previous study in a related *Sulfolobus* species, Sulfolobus solfataricus P2 (19). To dissect how *slaA* and *slaB* contribute, separately and/or jointly, to the S-layer assembly in detail, we used electron microscopy to observe the three derivatives of S. islandicus RJW004 harboring an in-frame deletion of either *slaA* (Δ*slaA*), *slaB* (Δ*slaB*), or both genes (Δ*slaAB*) (26) (see Materials and Methods).

**FIG 1:**
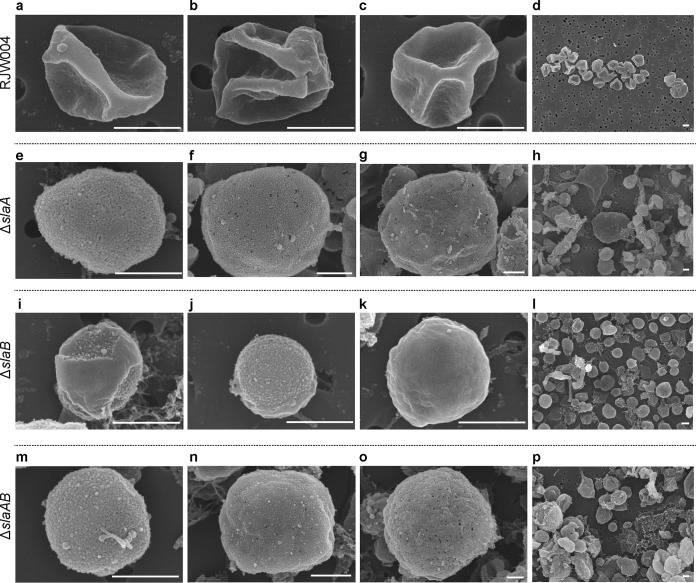
SEM analysis of wild-type RJW004 and S-layer gene knockout strains. Representative scanning electron micrographs show the wild-type cell with intact outermost layer (a to c), Δ*sla*A mutant cell with no outermost layer (e to g), Δ*slaB* mutant cell with partial (i), no (j), and intact (k) outermost layers, and Δ*slaAB* mutant cell with no S-layer (m to o). Overview SEM images (d, h, l, and p) show the varied cell morphologies, as indicated. Scale bar, 500 nm. Additional SEM images are provided in Fig. S2 (available at FigShare [https://doi.org/10.6084/m9.figshare.8285423]).

Figure 1a to d and Fig. S2a show scanning electron microscopy (SEM) images of the wild-type strain RJW004. We observed a regular lattice structure, characteristic of the S-layer, which covers the whole cell surface, making it look smooth. These cells show the irregular lobed morphology typically observed for *Sulfolobus* species in SEM and thin-section transmission electron microscopy (TEM) images (27, 28). In contrast, this outermost lattice layer was not observed in the Δ*slaA* (Fig. 1e to h and Fig. S2b) and Δ*slaAB* mutant cells (Fig. 1m to p and Fig. S2d) (26). For the Δ*slaB* mutant strain, most cells showed the outermost layer to be peeled off partially (Fig. 1i and Fig. S2c) or completely (Fig. 1j and Fig. S2c) from the cell surface although cells with an intact outermost layer were also rarely observed (Fig. 1k). These data further support the idea that SlaB functions as a stalk to stabilize and tether SlaA to the cell membrane but suggest that other proteins may serve this function as well. All mutant cells also showed an unfolded or round morphology, markedly different from that of wild-type cells (Fig. 1 and Fig. S2). The morphology change from lobed to round is consistent with the firm anchoring of the SlaA protein layer in the membrane being necessary for the rigidity that causes the irregular shape observed in SEM. This observation was also seen in thin-section TEM micrographs (26) as well as in S. solfataricus with a structurally unsound cell envelope under high-pressure freezing thin-section TEM (23). The cell sizes in Δ*slaA* and Δ*slaAB* mutants varied significantly, ranging from 0.3 to 6.8 μm (*n* = 432) and 0.2 to 6.0 μm (*n* = 667), respectively, compared to that of wild-type cells (0.4 to 1.7 μm; *n* = 778). Around 21% of cells in the Δ*slaA* mutant and 17% of cells in the Δ*slaAB* mutant were larger than the wild-type cell size range and very few (∼0.9% for the Δ*slaA* mutant and ∼0.8% for the Δ*slaAB* mutant) were below this range. The Δ*slaB* mutant cells varied in size between 0.1 and 1.6 μm (*n* = 646), with approximately 99% of cells in the wild-type cell size range.

**FIG 2:**
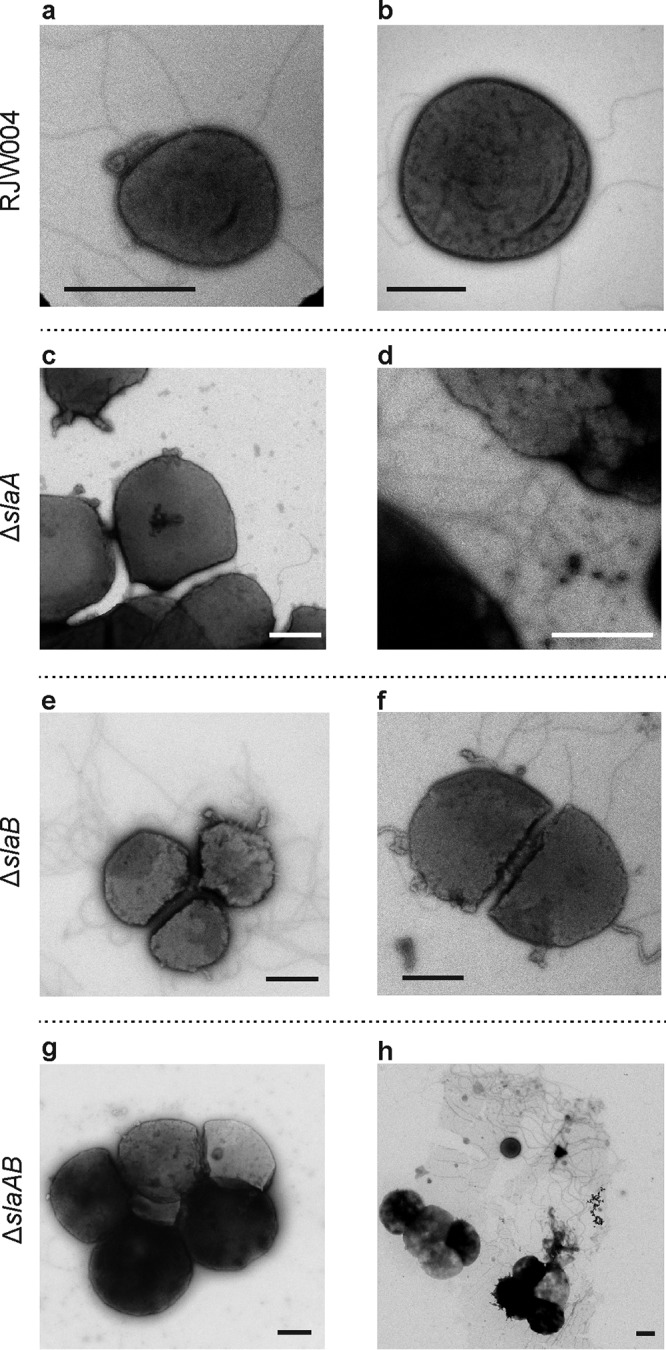
TEM analysis of the wild-type RJW004 and S-layer gene knockout mutants showing cell surface appendages. Shown are representative transmission electron micrographs of the wild-type and mutant cells, as indicated. Scale bar, 1 μm.

In support of our SEM analysis, conventional whole-cell TEM analysis revealed that an intact outermost layer was observed in the wild-type cells (see Fig. S3a and b, available at FigShare [https://doi.org/10.6084/m9.figshare.8285423]) but not in the Δ*slaA* (Fig. S3c and d) or Δ*slaAB* (Fig. S3g and h) mutant cells. A partial or discontinuous outermost layer was frequently observed in the Δ*slaB* mutant cells (Fig. S3e and f), demonstrating that SlaA was present on the outside of the cell but was fragile and unstable and, thus, was easily detached from the cytoplasmic membrane following TEM sample preparation without the support provided by the SlaB stalk. These observations are in agreement with those obtained recently from our thin-section TEM experiments (26). Interestingly, it is clear that cell surface appendages are expressed with visible pili and archaellum in all mutants (Fig. 2).

### M164_1049, a homolog of SlaB, does not functionally replace SlaB but likely assists SlaB to stabilize the outermost layer SlaA.

Electron microscopy (TEM, thin-section TEM, and SEM) analyses, presented both here and in a previous study (26), revealed that a partial SlaA was retained around the cell in the Δ*slaB* mutant, raising a question of whether there are homologs of SlaB that could fulfill the SlaB function. Using the SlaB protein sequence as a query, we previously identified a protein, M164_1049, annotated as a hypothetical protein in the genome, which shared 53% amino acid identity with SlaB with 42% query coverage (Fig. S4) (26). We next investigated whether M164_1049 plays a role in S-layer retention and particularly asked whether M164_1049 could partially or fully complement SlaB. To this end, we introduced a Δ*M164_1049* allele into the wild-type RJW004 and Δ*slaB* mutant strains using a recently developed microhomology-mediated gene inactivation (MMGI) strategy (29), which resulted in the single Δ*M164_1049* and double Δ*slaB* Δ*M164_1049* mutants, respectively. The *M164_1049* deletion in mutant strains was confirmed by PCR analyses with flanking and internal primer sets (Fig. S5a and b). The growth profiles of Δ*M164_1049* and Δ*slaB* Δ*M164_1049* mutants have no significant differences from the parental RJW004 and Δ*slaB* strains, respectively, in that they exhibited comparable growth rates and could reach approximately the same terminal optical densities at 600 nm (OD_600_) (Fig. S5c and d).

**FIG 3:**
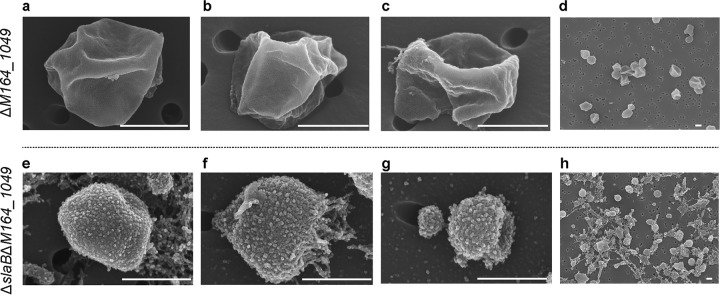
SEM analysis of the Δ*M164_1049* and Δ*slaB* Δ*M164_1049* mutant cells. Scale bar, 500 nm. Additional SEM images can be found in Fig. S6 (available at FigShare [https://doi.org/10.6084/m9.figshare.8285423]).

SEM images showed that the Δ*M164_1049* strain has a cell morphology very similar to that of its parental strain RJW004 (Fig. 3a to d; see also Fig. S6a available at FigShare [https://doi.org/10.6084/m9.figshare.8285423]). In contrast to Δ*slaB* mutant cells, a partial coat of SlaA is not observed in the Δ*M164_1049* mutant cells. The Δ*slaB* Δ*M164_1049* mutant appears to lack an S-layer (Fig. 3e to g), similar to the Δ*slaA* and Δ*slaAB* mutants. In the Δ*slaB* Δ*M164_1049* mutant, an increased amount of debris was observed (Fig. 3h and Fig. S6b), which might correspond to unbound SlaA and other extracellular components. The Δ*slaB* Δ*M164_1049* mutant cells may also have been susceptible to physical damage occurring in the process of SEM sample preparation. We found that about 33% of cells (*n* = 684) in the Δ*slaB* Δ*M164_1049* mutant are less than 0.5 μm in diameter whereas only 0.5% of cells (*n* = 778) in that range are found in the wild type. The conventional whole-cell TEM analysis revealed that an intact and smooth outermost layer, i.e., SlaA, was consistently observed to encompass the whole cell of the Δ*M164_1049* mutant (Fig. S7a and b), which was further validated by thin-section TEM (Fig. S7c and d). In agreement with the SEM analysis, the outermost layer was not observed in the mutant strain lacking both *slaB* and *M164_1049* genes in TEM images (Fig. S7e to h). These results suggest that M164_1049 may assist SlaB to firmly anchor the SlaA to the cytoplasmic membrane but is not redundant in its functioning in S-layer stabilization.

### Deficiency in SlaA results in large aggregates with variations in cell size.

**FIG 4:**
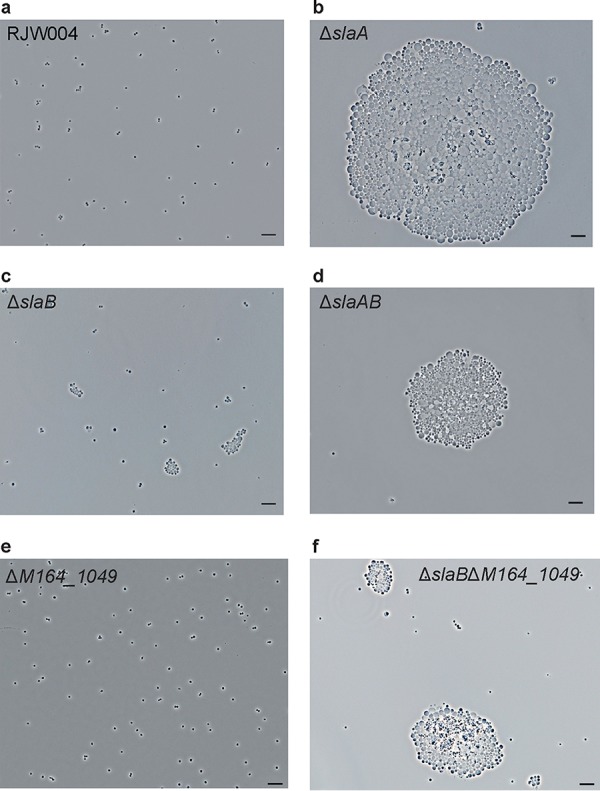
Phenotypic characterization of S-layer gene knockout mutants under a phase-contrast microscope. Representative phase-contrast microscopy images of cells of wild-type RJW004 and mutants are shown, as indicated. Five-microliter cell cultures were spotted on a cleaned microscope slide, covered with a coverslip, and then observed under a microscope. Scale bar, 10 μm.

**FIG 5:**
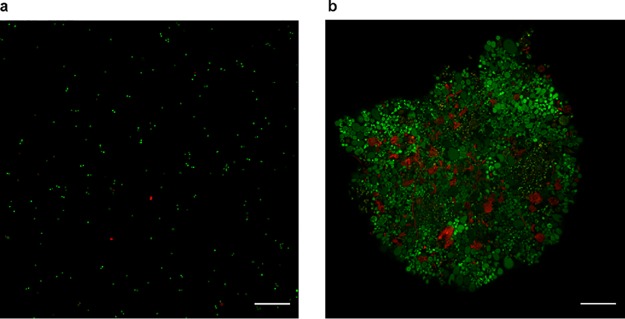
Live/dead fluorescence microscopy analysis of the wild-type RJW004 and the Δ*slaA* mutant. Cell cultures of RJW004 (a) and the Δ*slaA* mutant (b) were stained with a Live/Dead BacLight bacterial viability kit as described in Materials and Methods. Cells with intact and compromised membranes are labeled in green and red, respectively. Scale bar, 20 μm.

To characterize the phenotypes of these S-layer-deficient mutants, cell cultures taken from log-phase growth were observed under a light microscope. In contrast to our observations of the wild type (Fig. 4a), we found that the cells predominantly form bulky clumps in the Δ*slaA* mutant cultures, which generally contain hundreds or thousands of cells in a single clump or aggregate (Fig. 4b). We observed that the sizes of cells within the aggregates varied significantly in the Δ*slaA* mutant, ranging from ∼0.5 to 9 μm in diameter (*n* = 2,354). About 67% of cells in the Δ*slaA* mutant have a diameter greater than 2 μm whereas in the wild type only 5% of cells are found in this range (*n* = 2,978) (see Table S1, available at FigShare [https://doi.org/10.6084/m9.figshare.8285423]). We used live-dead fluorescence microscopy to see whether the aggregated cells in large clumps are alive or dead, and it revealed that most of the aggregated cells in the Δ*slaA* mutant are not permeable by propidium iodide (PI) (Fig. 5). This indicates that they still maintained an impermeable cell membrane, suggesting that the majority of the cells are alive. As with the Δ*slaA* mutant, a substantial number of large clumps containing large cells was observed in the Δ*slaAB* mutant cultures (Fig. 4d). The phenotypes, such as cell size and shape, of the Δ*slaA* and Δ*slaAB* mutants are very similar; however, the clumps in the Δ*slaAB* mutant are generally smaller than those of the Δ*slaA* mutant alone, suggesting a role for *slaB* in cell aggregation.

Deletion of the single *slaB* gene caused small aggregates in the cell cultures (Fig. 4c), and the cell size within the aggregates varied, though not as much as in the Δ*slaA* mutant, with a range of ∼0.5 to 4.0 μm (*n* = 2,863). We found that around 16% of cells in the Δ*slaB* mutant have a diameter greater than 2 μm (Table S1). No obvious cellular aggregation was seen in the single Δ*M164_1049* mutant cells (Fig. 4e), similar to observations in the wild-type RJW004 (Fig. 4a). Interestingly, the size of the aggregate increased in the Δ*slaB* Δ*M164_1049* double mutant (Fig. 4f) compared to that of the Δ*slaB* mutant. Large cellular clumps in the Δ*slaB* Δ*M164_1049* mutant at a scale similar to those in the Δ*slaA* and Δ*slaAB* mutants were observed but very rarely (data not shown).

The formation of aggregates in liquid culture is supported by the fact that although the Δ*slaA* mutant and wild-type cultures have comparable growth rates (26), we observed a decrease in OD_600_ values and CFU counts for the Δ*slaA* mutant (Fig. S8). Large aggregates would result in more light passing through the liquid culture when the optical density is measured, causing a lower OD_600_ value, as observed for the Δ*slaA* mutant. Aggregated cells may grow as one CFU in solid medium, consistent with the observation that there is a more than 10-fold reduction in the CFU count of the Δ*slaA* mutant in spotting assays (Fig. S8). We cannot exclude the possibility that the lower CFU count may be caused or contributed by other factors such as an increased sensitivity to cell processing procedures.

### S. islandicus cells deficient in S-layer exhibit a higher sensitivity to osmotic stress than the wild type but do not decrease in size.

**FIG 6:**
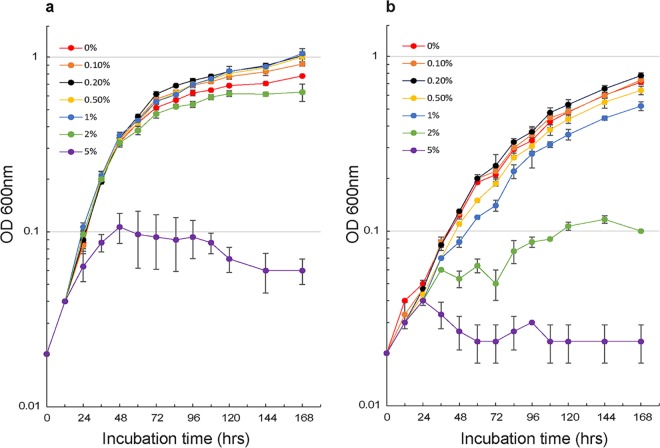
Growth profiles of wild-type RJW004 and Δ*slaA* mutant strains in dextrin-tryptone medium supplemented with different concentrations of sucrose. The wild-type strain RJW004 (a) and Δ*slaA* mutant (b) were cultivated at 78°C in the presence of 0%, 0.1%, 0.2%, 0.5%, 1%, 2%, and 5% sucrose solution. Cell growth was monitored for 7 days by measuring the OD_600_ of cell cultures. Error bars represent standard deviations.

In agreement with a theoretical study (14), we hypothesize that, without the S-layer to constrain cell expansion and counteract the turgor pressure, the cells will become distended. If large cells result from turgor pressure, expanding cells without the presence of an S-layer grown in medium with higher concentrations of extracellular solutes should relieve this pressure and result in smaller cell morphologies. To test this hypothesis, we grew wild-type RJW004 and Δ*slaA* mutant cells in liquid medium supplemented with increasing amounts of sucrose. The wild-type strain was able to grow in the presence of 2% (wt/vol) sucrose with a growth rate that was still comparable to that of cells that grew in sucrose medium with concentrations ranging from 0% to 1%; however, cell growth was severely impaired when cells were cultivated in 5% sucrose medium (Fig. 6a). In contrast, at above a 1% sucrose concentration, growth of the Δ*slaA* mutant was significantly delayed (Fig. 6b). We further examined cells after a 48-h incubation using live-dead fluorescence microscopy and found that the proportion of wild-type cells with permeable membranes remained at around 8% (*n* = 788) and 26% (*n* = 740) when cells were cultured in 2% and 5% sucrose medium, respectively (see Fig. S9a, available at FigShare [https://doi.org/10.6084/m9.figshare.8285423]). For the Δ*slaA* mutant, the estimated proportion of permeable cells increased by more than 50% and 90% in the presence of 2% and 5% sucrose, respectively; in particular, the membranes of relatively enlarged cells were more prone to be permeable at high concentrations of sucrose (Fig. S9b). Even under high-osmotic-stress conditions, impermeable cells in the Δ*slaA* mutant were significantly larger than wild-type cells (Fig. S9b), indicating that large cells observed in the Δ*slaA* mutant are not expanding due to turgor pressure alone. These results collectively showed that S. islandicus cells deficient in S-layer are more sensitive to hyperosmotic stress than wild-type cells.

### S. islandicus cells deficient in S-layer exhibit variations in DNA content.

**FIG 7:**
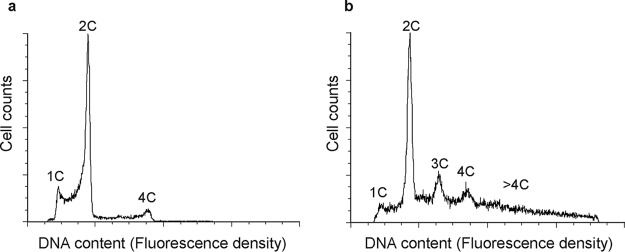
Flow cytometric profiles of the wild-type RJW004 (a) and Δ*slaA* mutant (b) strains. Cells from exponentially growing cultures were used. Cells with 1C (1 chromosome), 2C, 3C, and 4C DNA content in the fluorescence histograms are indicated.

*Sulfolobus* cells with a cell division defect often have an enlarged-cell-size phenotype (30, 31). To investigate whether the absence of the S-layer might affect cell division, wild-type RJW004 and Δ*slaA* mutant cells, taken from log-phase growth, were analyzed with flow cytometry. As shown in Fig. 7a, RJW004 exhibited a typical chromosomal pattern of the cell cycle, which was also observed in other *Sulfolobus* species (32, 33). However, in the Δ*slaA* mutant strain, the peaks in the fluorescence histogram corresponding to three copies of chromosome (3C) and four copies of chromosome (4C) as well as a long trailing tail became distinct, suggesting the existence of multiple chromosomes in the cells (Fig. 7b). It should be noted that the Δ*slaA* mutant cells tend to form big clumps in cultures, which interfere with interrogating the chromosome content of single cells using flow cytometry. Gating the single cells or including larger aggregates revealed that both have clear 3C and 4C peaks as well as a long tail with higher chromosome content (Fig. S10). We note that in contrast to the wild-type cells, Δ*slaA* cells contain a greatly reduced population in the process of replicating between the 1C (1 copy of chromosome) and 2C (2 copies of chromosome) peak (SSC-A/Alexa Fluor 488-A shown in Fig. S10). The cell size distribution of Δ*slaA* and Δ*slaB* mutants was further evaluated by fluorescence-activated cell sorting (FACS) analysis. We found that a substantial proportion of enlarged cells existed in the Δ*slaA* mutant; however, the size distribution profiles between the Δ*slaB* and wild type were hardly distinguishable (Fig. S11).

## DISCUSSION

The physiological and cellular functions of the archaeal S-layer had remained largely unexplored, likely due to experimental limitations or unfeasibility of creating viable S-layer-deficient mutants. Here, electron microscopy analyses of S. islandicus S-layer gene deletion mutants (26) provide strong and direct *in vivo* evidence in support of a so-called stalk-and-cap model of the S-layer proposed in *Sulfolobales* (9, 34). Notably, we identified and genetically characterized a novel S-layer-related gene, *M164_1049*, encoding an SlaB paralog. The predicted transmembrane helix (see Fig. S4, available at FigShare [https://doi.org/10.6084/m9.figshare.8285423]) present in both the N and C termini of M164_1049 suggests that M164_1049 has a membrane topological organization, implying that there is a potential connection between M164_1049 and the outermost layer (SlaA). Interestingly, the M164_1049 homolog in a related *Sulfolobus* species, S. solfataricus P2 (SSO1175, sharing 83% amino acid identity with 96% query coverage) has been shown to be an N-glycosylated protein (35) and biochemically characterized to function as a multidomain thermopsin-like protease (36). Collectively, our results show that M164_1049 serves as a secondary stalk and assists the primary stalk SlaB to anchor SlaA firmly to the cytoplasmic membrane. Given the broad distributions of M164_1049 within *Sulfolobales*, the role of M164_1049 in anchoring the SlaA lattice structure when SlaB is absent, though via a weak connection, should be considered when building a new model of the S-layer in *Sulfolobales*. These data show that our understanding of interactions between membrane proteins and the crystalline layer SlaA are only just beginning to be explored.

S. islandicus cells that are deficient in SlaA, either caused by a genetic deletion of the *slaA* gene from the chromosome (Δ*slaA* and Δ*slaAB* mutants) or by the physical absence of SlaA as in the Δ*slaB* Δ*M164_1049* mutant cells, form large aggregates composed of cells with increased diameters. The Δ*slaB* mutant also exhibits a high level of cellular aggregation (approximately 80% of cells are in aggregates that contain more than 4 individual cells), but the sizes of aggregates along with the cell diameters are smaller than those of the Δ*slaA* mutant. We proposed that the high level of cellular aggregation but of relatively small-size aggregates in the Δ*slaB* mutant may be due to a large proportion of cells that retained a partial outermost layer (SlaA), as revealed by electron microscopy analysis of the Δ*slaB* mutant. This viewpoint was further supported by the FACS analysis in which the cell size distribution of the Δ*slaB* mutant exhibits a pattern as similar to that of the wild type. Taken together, these data suggest that SlaA, not SlaB, is the key determinant of cellular aggregation and cell morphology in S. islandicus though the exact contribution of SlaB to these phenotypes remains to be investigated.

We suggest two models to explain the combination of large cell sizes within aggregates and surprisingly variable odd numbers of chromosomes observed in the Δ*slaA* mutant: cell fusion and a cell division defect. In the cell fusion model, the SlaA-deficient cells aggregate, possibly because of interactions between outer membrane proteins or attraction between hydrophobic membranes themselves when cells are not protected by the S-layer outer shell. Within aggregates, cells fuse, resulting in an increase in cell size. As the cells continue to grow over time, a wide range of cell diameters would be created, as observed in the Δ*slaA* mutant. We suspect that this type of hypothesis alone would result in mutants with a greater increase in even-number chromosome copy numbers due to the higher chance of contact between the cells that are in an extensive G_2_ stage during the *Sulfolobus* cell cycle (33). The distinct 3C peak observed in flow cytometry analysis may result from 1C cells are fusing with 2C cells before they replicate the chromosome. Alternatively, cells without the S-layer could be deficient in cell division and/or cell cycle checkpoints in DNA replication. The larger cells observed in the Δ*slaA* mutant can be explained by a cell division defect as a similar phenotype of >2C chromosomes has been observed in S. islandicus cells with a deletion in the cell division-related component ESCRT (endosomal sorting complexes required for transport)-III-1 (37); however, in this study the Δ*escrt-III-1* mutant cells formed chains and did not form aggregates with increased cell size to the extent that we observed. In addition, the enlarged cells with sizes increasing up to 2- to 3-fold have also been observed in an Sulfolobus cidocaldarius strain deficient in ESCRT-III-3 (CdvBIII), a paralog of ESCRT-III that was shown to play an auxiliary role in cell division (30). Though S-layer gene deletion mutants have been created and characterized in several bacteria (3–5, 38, 39), cell division defect phenotypes in those mutants have rarely been reported (3). In the cell division defect scenario, the observation of aggregates would represent clusters of replicating cells that have not fully divided. Cell populations with an obvious 3C peak in the Δ*slaA* mutant can be explained by one daughter cell rereplicating while the other does not before the cell division occurs. This is very unusual for *Sulfolobus* cells in which the cell cycle is tightly regulated (40). In addition, we observed a decreased number of cells transitioning between 1C and 2C (Fig. S10, available at FigShare [https://doi.org/10.6084/m9.figshare.8285423]), suggesting the possibility that cells were no longer dividing to 1C but replicating before the division was completed.

The cell fusion and cell division hypotheses are clearly not mutually exclusive, and both cell fusion and irregularities in the cell cycle may be linked to S-layer function. Addressing these questions will allow for a more complete understanding of the S-layer function in *Sulfolobus* and archaeal cell biology as a whole as well as of interactions with fundamental processes such as chromosome partitioning.

## MATERIALS AND METHODS

### Strains and growth conditions.

The strains and plasmids used in this study are listed in Table S2 (available at FigShare [https://doi.org/10.6084/m9.figshare.8285423]). The E. coli Top10 strain (Invitrogen, USA) was used to maintain and propagate plasmid DNA and grown in Luria-Bertani (LB) liquid medium at 37°C. When required, 100 μg/ml ampicillin was supplemented in LB agar plates to select ampicillin-resistant clones. S. islandicus RJW004, the genetic host used in this study, was originated from wild-type isolate S. islandicus M.16.4 (41), which carried in-frame deletions of *pyrEF*, *lacS*, and *argD* (42). S. islandicus strains were cultured at 76 to 78°C without shaking in dextrin-tryptone liquid medium (0.2% [wt/vol] dextrin, 0.1% [wt/vol] tryptone, pH 3.3), as described previously (29). When necessary, 20 μg/ml uracil and/or 50 μg/ml agmatine was added. S. islandicus single colonies were isolated on Gelrite-solidified plates, formulated as described previously (42), via an overlay cultivation approach (43). Plates were put into sealed plastic bags and cultivated for 10 to 20 days at 76 to 78°C. Cell growth was monitored by optical density measurements at 600 nm (OD_600_) with a CO8000 cell density meter (WPA, Cambridge, United Kingdom).

### RT-PCR analysis.

Ten milliliters of cell cultures taken from exponentially growing strains was harvested and lysed with 1 ml of TRIzol reagent (Invitrogen, USA). Total RNA was extracted and isolated with a PureLink RNA Minikit (Thermo Fisher Scientific, USA) and then cleaned and concentrated with an RNA Clean & Concentrator-25 kit (Zymo Research, USA). To exclude potential DNA contamination, the total RNA (∼2 μg) was treated with 2 μl of amplification-grade DNase I (1 U/μl; Invitrogen, USA). Approximately 500 ng of DNase I-treated total RNA was reverse transcribed using 2 μM gene-specific reverse primers and 1 U of SuperScript IV reverse transcriptase (Thermo Fisher Scientific, USA) according to the manufacturer’s guidelines. Two microliters of synthesized cDNA was used as a template in a standard PCR amplification reaction performed in a thermocycler (Bio-Rad, USA), using Phusion High-Fidelity DNA polymerase (NEB, USA) and the gene-specific primers binding the regions inside the *slaA* and *slaB* genes (Table S3). Genomic DNA and total RNA were used as templates for positive and negative controls in PCR amplification, respectively. The resulting PCR products were separated by electrophoresis with a 1.2% (wt/vol) Tris-acetate EDTA (TAE) agarose gel.

### Genome sequencing.

Genomic DNA of S. islandicus cells was extracted using a DNeasy Blood & Tissue kit (Qiagen, USA) according to the manufacturer’s instructions. Sequencing libraries of genomic DNA from wild-type RJW004 and the Δ*slaA*, Δ*slaB*, and Δ*slaAB* mutants were constructed with a Nextera XT DNA Library Preparation kit (Illumina, USA) individually. Libraries were sequenced using HiSeq MMD (250-bp paired-end). The raw sequencing reads were processed with a quality filtering and then mapped to the reference genome of S. islandicus M.16.4 (the ancestor strain of RJW004) with breseq (44) using default settings to identity the mutations. The mutations that occurred in Δ*slaA*, Δ*slaB*, and Δ*slaAB* compared to the sequence of their parental strain RJW004 are summarized in Table S4. Besides the expected deletions found in the S-layer gene region, 3 to 7 independent mutations were found; however, none of these mutations occurred in the genes known to be related to surface layer. One of the Δ*slaAB* isolates (Δ*slaAB-6*) harbored a 363-bp deletion in M164_1772 annotated as a hypothetical protein. Although the function of M164_1772 is unclear, it should not contribute to the phenotypes as observed in the Δ*slaAB* mutant because characterizations of another Δ*slaAB* isolate without any *M164_1772* mutation (Δ*slaAB-2*) demonstrated the same phenotype as that of the Δ*slaAB-6* mutant (this study) under electron microscopes (data not shown).

### Construction and verification of the *M164_1049* gene deletion mutants in S. islandicus.

Deletion of *M164_1049* in the genetic background of RJW004 and RJW012 (see Table S2, available at FigShare [https://doi.org/10.6084/m9.figshare.8285423]) was accomplished by replacing it with an arginine decarboxylase expression cassette (*StoargD*) (the *argD* gene from Sulfolobus tokodaii), using a recently developed MMGI approach (29). Agmatine prototrophic (ArgD^+^) transformants were selected on dextrin-tryptone plates containing 20 μg/ml of uracil but lacking agmatine. The *M164_1049* deletion mutants were verified by a colony PCR procedure described previously (42) with two primer sets: (i) a flanking primer set annealing the outside region of the target gene and (ii) an internal primer set that specifically bound the inside region of the target gene. The resulting PCR products were treated with an ExoSAP-IT PCR Product Cleanup reagent kit (Thermo Fisher Scientific, USA) and then sequenced to confirm the insertion of the *StoargD* marker cassette in the desired position. S. islandicus mutant strains were purified at least once by an overlay plating method (43) and then used for further studies.

### Electron microscopy.

Samples used for conventional TEM analysis were prepared as follows. Carbon type B grids (200-mesh; TED Pella, Inc.) were placed on 20-μl droplets of S. islandicus cell cultures for 3 min. The residual cell cultures on the edge of grids were adsorbed with Whatman filter paper and washed with degassed water, and then the grids were negatively stained with 2% (wt/vol) uranyl acetate for 15 to 30 s. Samples used for thin-section TEM analyses were essentially prepared as described by Bautista et al. (45) with minor modifications (26). The grids were observed with a Philips CM200 transmission electron microscope operated at 120 kV. Images were taken using a Peltier-cooled Tietz TVIPS 2,000-by 2,000-pixel charge-coupled-device (CDD) camera, and processed with EM-MENU software. For SEM analysis, 8 to 10 ml of cell cultures taken from the mid-log phase was filtered through a 0.2-μm-pore-size filter (Whatman, USA) with a 10-ml syringe and then fixed at 4°C for 4 h in a fixative solution (2.0% paraformaldehyde and 2.5% glutaraldehyde in 0.1 M sodium cacodylate buffer, pH 7.4). Afterwards, the fixation buffer was removed and replaced with a rinse buffer (0.1 M sodium cacodylate buffer, pH 7.4). Samples were gently shaken for 10 min and then gradually washed in 37%, 67%, 95%, and 100% (vol/vol) ethanol for 10 min each. Following the last step, the samples were washed twice more with the 100% ethanol, and the filters were critical point dried within 48 h using an Autosamdri-931 critical point dryer (Tousimis). To observe the samples, filters were sputter coated with Au-Pd and then imaged with a Hitachi S-4800 high-resolution SEM at 10 kV at various magnifications.

### Phase-contrast microscopy.

S. islandicus strains were grown to the mid-log phase. Five microliters of cell cultures was spotted on a cleaned microscope slide using a blunted 20-μl pipette tip in order to avoid potential disruption of the large aggregates formed in the S-layer gene deletion mutants. A coverslip was added immediately, and then the slide was observed on a Zeiss Axiovert 200M microscope with a 63×/1.40 oil objective. Images were captured using a Zeiss Axiocam 506 microscope camera and visualized with Zeiss Zen imaging software.

### Flow cytometry.

Three hundred microliters of S. islandicus cells was fixed with 700 μl of ice-cold absolute ethanol, and the mixture was then briefly vortexed. Fixed samples were stored at 4°C for at least 12 h. Afterwards, the fixed cells were centrifuged at 13,000 rpm for 5 min and resuspended in 1 ml of cold Tris-MgCl_2_ buffer (10 mM Tris-HCl, pH 7.5, 10 mM MgCl_2_). Then the samples were precipitated and resuspended again in 300 μl of Tris-MgCl_2_ buffer containing 2 μg/ml of Sytox Green nucleic acid stain (Thermo Fisher Scientific, USA) and 100 μg/ml of RNase A (DNase and protease free; Thermo Fisher Scientific, USA). After at least a 30-min incubation on ice in the dark, samples were analyzed using an LSR II (BD Biosciences, USA) flow cytometer, with a 488-nm (50 mW) blue laser as excitation light and a 530/30-nm band pass filter. A total of 100,000 cell counts for each sample were collected. Flow cytometry data were processed and analyzed with De Novo FCS Express 6 software. The cell population was gated with three steps as described in the Fig. S10 (available at FigShare [https://doi.org/10.6084/m9.figshare.8285423]) to obtain a final DNA content histogram.

### Live-dead staining and microscopy.

RJW004 and the Δ*slaA* mutant strain were grown to mid-log phase. For the wild-type RJW004, 1 ml of cultures was transferred to a 1.5-ml microcentrifuge tube and then centrifuged at 10,000 rpm for 5 min to pellet. The pellet was resuspended with 1 ml of dextrin-tryptone (1×) medium to wash. Centrifugation was repeated once, with the resulting cell pellet then resuspended in 500 μl of dye solution. For the Δ*slaA* mutant cells, to preserve the aggregate phenotype, a total of 5 microcentrifuge tubes containing 1 ml of cultures were allowed to settle for 15 min to collect cells at the bottom of the tubes. Cells of all 5 microcentrifuge tubes were then combined, with supernatant removed. The remaining cells were resuspended in 1 ml of fresh dextrin-tryptone liquid medium. Cells were allowed to settle for an additional 10 min. Supernatant was removed, and the cells were gently resuspended in 500 μl of the dye solution, as per protocol (3 μl of 1:1 dye components A and B per 1,000 μl of dextrin-tryptone medium) (L7007 Live/Dead BacLight Bacterial Viability kit; Invitrogen, USA). Cells were covered and incubated at room temperature in darkness for at least 15 min before viewing. Five microliters of stained cells was then spotted directly on a microscope slide using a blunted 20-μl pipette tip, immediately covered with a coverslip, and observed on a Zeiss LSM 710 confocal microscope with a 63×/1.40 oil M27 objective at excitation wavelengths of 561 nm (for PI detection) and 488 nm (for Syto 9 detection) using Zeiss Zen software.

### Osmotic pressure test.

For the osmotic stress assay, a 50% sucrose solution was added to 45 ml of dextrin-tryptone liquid medium, making the final concentrations (wt/vol) of sucrose 0%, 0.05%, 0.10%, 0.2%, 0.5%, 1.0%, 2.0% and 5.0%. Proper volumes of S. islandicus cells were transferred into the medium to make an initial OD_600_ of 0.02. Three biological replicates were established for each treatment. Cells were grown at 78°C without shaking, and their growth was monitored for 7 days by measuring the OD_600_ usually every 12 or 24 h. Cell cultures after a 48-h incubation were examined with a Zeiss LSM 710 confocal microscope.

### Data availability.

The genome sequence data of the reference strain S. islandicus M.16.4 were previously published (GenBank accession no. CP001402.1). The raw sequencing data of the parental strain S. islandicus RJW004 and its derived S-layer deletion mutants are available upon request.
